# Resolution of ventricular tachycardia and cardiac inflammation following treatment in chronic Chagas disease

**DOI:** 10.1016/j.hrcr.2024.08.015

**Published:** 2024-08-16

**Authors:** Aneeq Malik, Michael Jiang, Gisele Muñoz, Sheba Meymandi, Jason S. Bradfield, Justin Hayase

**Affiliations:** 1 Center of Excellence for Chagas Disease, Olive View UCLA Medical Center, Sylmar, CA; 2 UCLA Cardiac Arrhythmia Center, UCLA Medical Center, Los Angeles, CA

**Keywords:** Chagas cardiomyopathy, Ventricular arrhythmia, Benznidazole, Amiodarone, 18-FDG PET-CT

**Key Teaching Points**

• Role of inflammation in chronic Chagas disease (CD): Chronic CD-related cardiomyopathy (CM) can show inflammation on 18F-fluoro-2-deoxy-d-glucose positron emission tomography, which may contribute to ventricular arrhythmias. This highlights the need for further investigation into inflammation's role in CD-related CM.
• Benznidazole's impact on inflammation and arrhythmias: Antiparasitic treatment with benznidazole can resolve inflammation in CD-related CM, as indicated by reduced 18F-fluoro-2-deoxy-d-glucose positron emission tomography uptake. This reduction correlates with decreased or resolved ventricular arrhythmias, suggesting a potential benefit of benznidazole treatment.
• Implications for clinical guidelines: Current guidelines restrict antiparasitic treatment to early stages of CD-related CM. Evidence from this case suggests that patients with ventricular arrhythmias and PET-detected inflammation might also benefit, indicating a potential expansion of treatment candidacy.

## Introduction

Chagas disease (CD), caused by the parasite *Trypanosoma cruzi*, is the third most common parasitic disease and one of the leading causes of death in Latin America.^1^ It affects multiple organ systems but predominantly impacts the cardiac system, leading to cardiomyopathy (CM); conduction abnormalities; and ventricular arrhythmias, such as ventricular tachycardia (VT) or ventricular fibrillation. Although active inflammation is well-documented in acute infection,^2^ its role in chronic CD-related CM remains unclear. Patients with CD-related CM are at an increased risk of developing monomorphic VT and have higher mortality rates compared with those with other forms of nonischemic CM.^3^ It has been postulated that chronic inflammation may serve as a nidus for arrhythmias in these patients.^4^ Positron emission tomography computed tomography (PET-CT) has been suggested as a modality to localize this chronic inflammation, potentially linking it to the occurrence of VT. Although the BENEFIT trial indicated that treatment did not significantly reduce cardiac mortality in patients with CD-related CM, the role of treatment in patients with CD-related CM, sustained ventricular arrhythmias, and inflammation on PET-CT has not been well described.^5^ Here, we present the case of a patient with CD who experienced recurrent episodes of sustained VT, underwent antiparasitic treatment with benznidazole, and subsequently had resolution of inflammatory changes on PET-CT and no further ventricular arrhythmias.

## Case report

**Figure 1:**
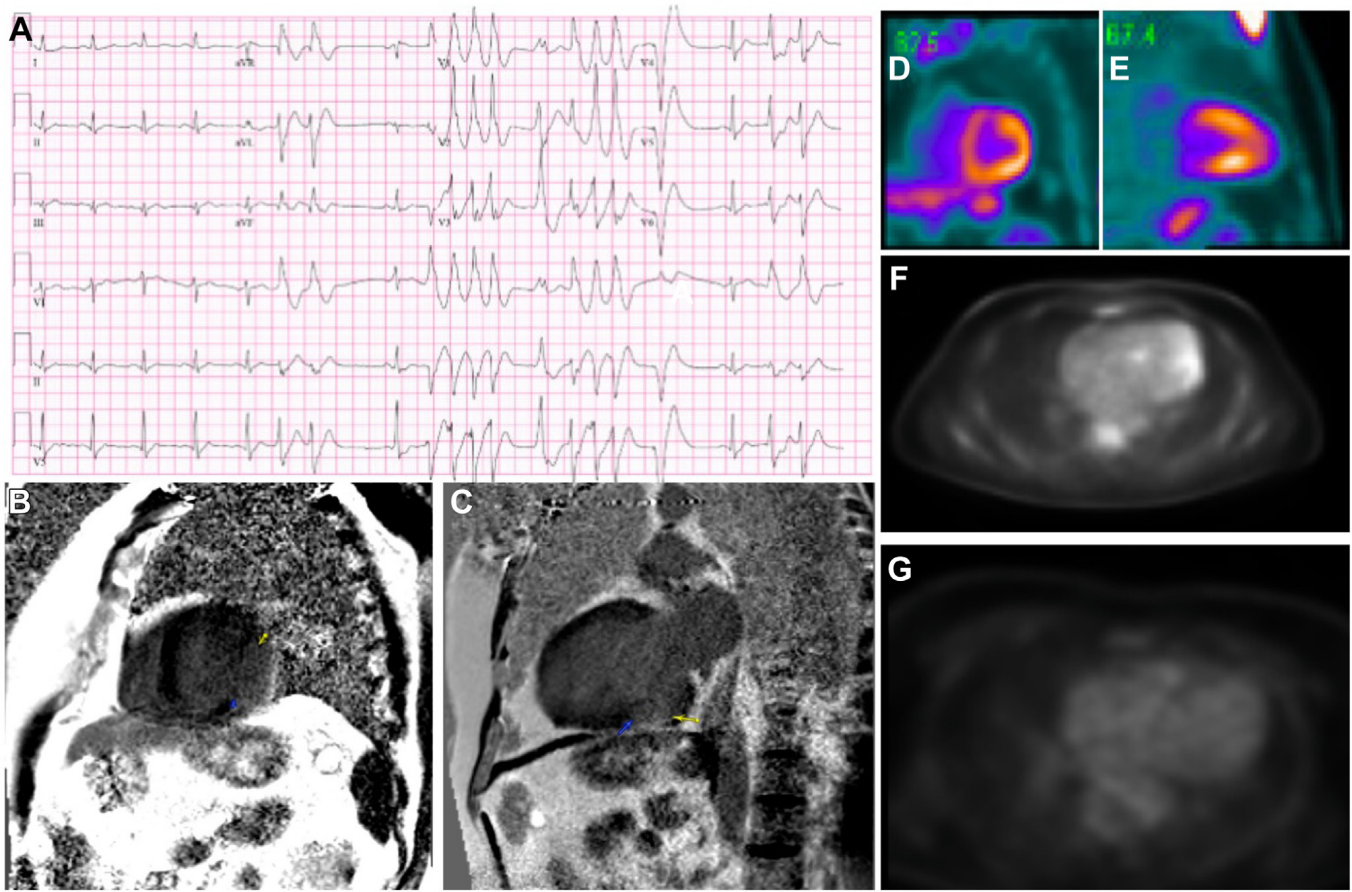
**A:** Electrocardiogram demonstrating nonsustained ventricular tachycardia with a likely inferolateral exit site. **B, C:** Pretreatment cardiac magnetic resonance images demonstrating late gadolinium enhancement in the basal inferior and inferolateral walls (yellow and blue arrows). **D–F:** Pretreatment cardiac 18F-fluoro-2-deoxy-d-glucose positron emission tomography-computed tomography (18-FDG PET-CT) scans showing diffuse FDG uptake most prominent in the basal-mid inferolateral and basal inferior walls. **G:** Post-treatment cardiac 18-FDG PET-CT scan showing absence of abnormal FDG uptake throughout the myocardium.

A 42-year-old man, originally from Mexico, with a history of hypertension presented to another hospital initially after experiencing an out-of-hospital cardiac arrest due to VT and ventricular fibrillation. He was successfully resuscitated and underwent left heart catheterization, showing no obstructive coronary artery disease. He underwent cardiac magnetic resonance imaging, which revealed a dilated left ventricle, late gadolinium enhancement, and hypokinesis in the basal inferolateral portion of the left ventricle (Figure 1B and 1C), with an estimated left ventricular ejection fraction of 36%. A single-chamber Biotronik (Berlin, Germany) implantable cardioverter defibrillator (ICD) with an atrial dipole was placed for secondary prevention, and he was discharged on guideline-directed medical therapy, which included a beta blocker.

Eighteen months later, he presented to another hospital following multiple ICD shocks for episodes of ventricular fibrillation and VT. He was started on amiodarone but had incessant VT storm, ultimately requiring intravenous infusions of amiodarone, lidocaine, and esmolol. The patient was transferred to a tertiary referral center for consideration of advanced heart failure therapies or epicardial catheter ablation. A 12-lead electrocardiogram capturing nonsustained VT suggested an inferolateral left ventricular VT exit site (Figure 1A). A device interrogation revealed that 106 appropriate ICD shocks and 17 episodes of appropriate antitachycardia pacing were delivered over the span of 3 days before clinically improving on multiple antiarrhythmic medications. A cardiac 18F-fluoro-2-deoxy-d-glucose (18-FDG) PET-CT was performed, which revealed large areas of diffuse, moderate to intense FDG activity, most prominently involving the basal inferior and basal to mid inferolateral left ventricular wall segments (Figure 1D–1F), which correlated with the VT exit site suggested by his 12-lead electrocardiogram. The 18-FDG PET-CT findings raised concern for an inflammatory CM. Because of the possibility of cardiac sarcoidosis, the patient was discharged on a high-dose steroid taper in addition to amiodarone.

Both screening and then later confirmatory serologic testing for *T cruzi* resulted positive and the patient was suspected to have Chagas CM. The patient was then referred to a Chagas Center of Excellence to establish care. Because of his reduced left ventricular ejection fraction, he was not considered to be a candidate for antiparasitic therapy, in accordance with current clinical trial evidence and expert recommendations.^5^^,^^6^ He did not have recurrent ventricular arrhythmias requiring ICD therapies until 10 months later, when he was admitted to another hospital for multiple ICD shocks for slow VT episodes. Device interrogation revealed 2 sustained slow VT episodes that received 8 antitachycardia pacing attempts before requiring 2 shock therapies in total. There was another episode of sustained slow VT that was below the VT therapy zone. He was started on mexiletine, in addition to his regimen of amiodarone and metoprolol. A repeat transthoracic echocardiogram revealed a new aneurysmal segment of the basal inferior myocardium. A referral for epicardial or endocardial catheter ablation of VT was made, but this was not immediately available because of insurance limitations. Considering these findings and recurrent VT episodes, he was given antiparasitic treatment empirically to help prevent progression of disease with benznidazole 100 mg orally twice daily for 60 days.

Approximately 1 month following benznidazole treatment, the patient had 1 episode of VT, which terminated successfully with 1 appropriate ICD shock. Four months after antiparasitic treatment, he had 2 additional episodes of VT that were terminated successfully and appropriately by antitachycardia pacing. A repeat 18-FDG PET-CT 19 months from the initial diagnosis and 6 months from completion of antiparasitic treatment demonstrated total absence of abnormal FDG activity (Figure 1G). Amiodarone was discontinued at this time because of thyroid toxicity.

**Figure 2:**
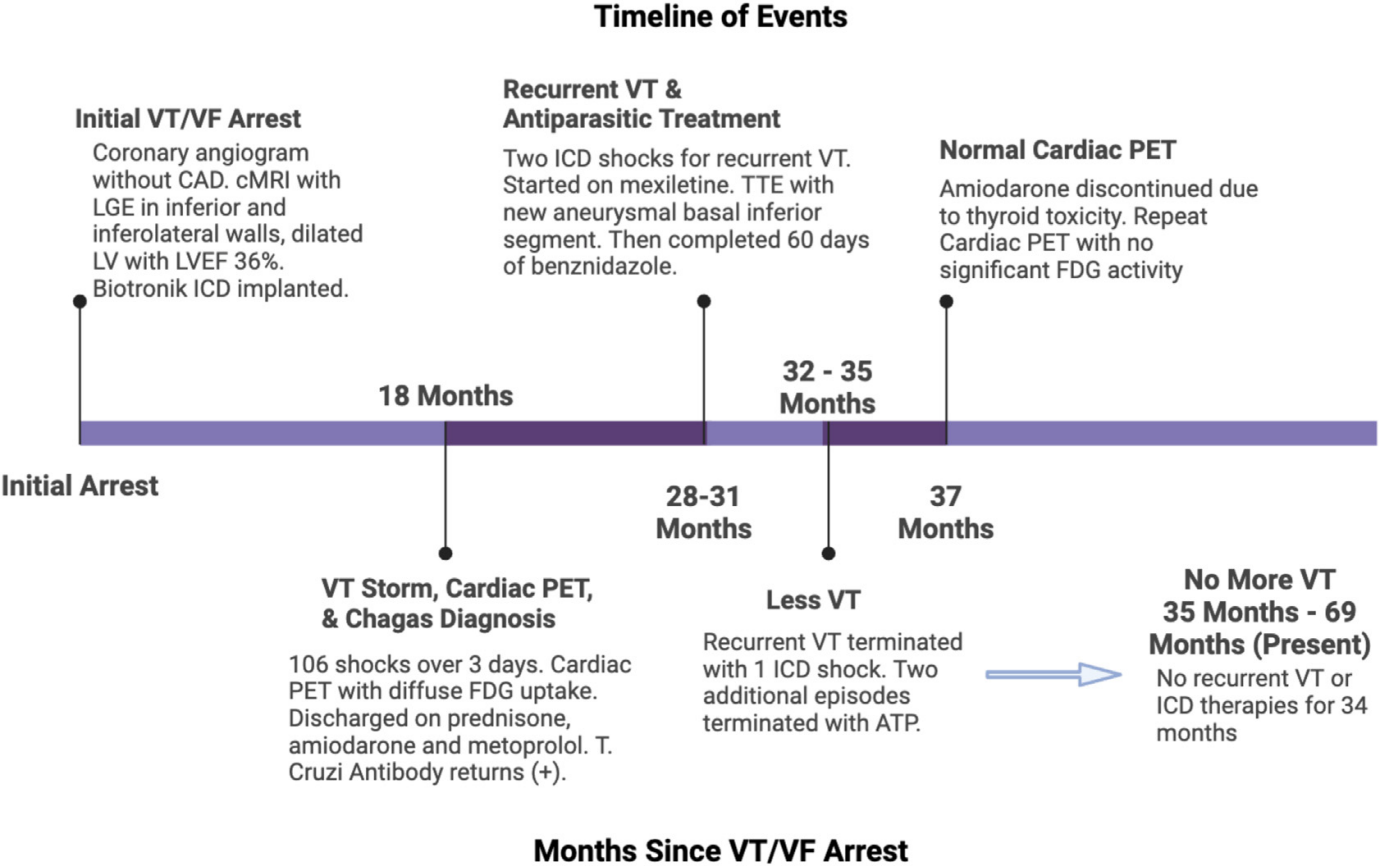
Timeline summarizing important clinical events. ATP = antitachycardia pacing; CAD = coronary artery disease; cMRI = cardiac magnetic resonance imaging; FDG = fluoro-2-deoxy-d-glucose; ICD = implantable cardioverter defibrillator; LGE = late gadolinium enhancement; LV = left ventricular; LVEF = left ventricular ejection fraction; PET = positron emission tomography; T. Cruzi = *Trypanosoma cruzi*; TTE = transthoracic echocardiogram; VF = ventricular fibrillation; VT = ventricular tachycardia.

The patient has since remained free of sustained ventricular arrhythmias through at least 34 months of additional follow-up, despite discontinuation of amiodarone (Figure 2). Given the lack of recurrent VT episodes, catheter ablation of VT was ultimately deferred. He continues to follow-up in both cardiology and Chagas center clinics.

## Discussion

The major findings of the present case are as follows:

1. Areas of inflammation, as noted by regions of abnormal uptake on 18-FDG PET-CT imaging, may contribute to clinical VT in patients with chronic CD-related CM.
2. Antiparasitic treatment with benznidazole in patients with chronic CD-related CM may be associated with resolution of areas of inflammation, as seen on 18-FDG PET uptake.
3. This decrease in inflammation may be associated with a reduction in burden or even resolution of clinical ventricular arrhythmias, potentially expanding the population that may derive benefit from antiparasitic treatment.

*T cruzi* causes an acute illness that can be cured with antiparasitic treatment. However, the role of antiparasitic treatment in chronic CD-related CM has been a subject of debate. Current guidelines from the World Health Organization limit antiparasitic treatment for patients with CD-related CM to those in the early stages.^6^ In the BENEFIT trial, patients with CD-related CM treated with benznidazole were observed to have a decreased parasitic burden, but no decrease in the primary composite outcome, which included death, resuscitated cardiac arrest, and sustained VT. However, the event rates for sustained VT in subgroup analysis were low (ie, 2.3%–2.9% in each arm).^5^ The use of 18-FDG PET-CT to evaluate for the presence of inflammation was also not used to guide management in the BENEFIT trial.

Prior case reports have postulated that persistent cardiac inflammation may also contribute to the increased risk of sudden cardiac death by providing a substrate for VT.^4^ It was suggested that it may provide a trigger for re-entry within adjacent scar tissue or be a substrate for focal VT. Based on current guidelines, patients with CD are not routinely screened for evidence of cardiac inflammation.

In the present case, an 18-FDG PET-CT was able to demonstrate active inflammation in a patient with nonischemic CM, recurrent ventricular arrhythmias, and serologies positive for *T cruzi*. Although an alternative diagnosis, such as cardiac sarcoidosis, was possible, these arrhythmias persisted, despite the titration of multiple different antiarrhythmic medications and steroid therapy, including amiodarone, mexiletine, and metoprolol. Electrical quiescence occurred only after the patient was given empiric antiparasitic treatment with a course of benznidazole, which was thereafter sustained in spite of discontinuation of amiodarone. Furthermore, there was a temporal relationship between antiparasitic treatment and interval resolution of inflammatory changes on follow-up 18-FDG-PET-CT. This case lends further evidence to the hypothesis that persistent parasite burden in chronic CD-related CM may be a trigger for inflammation, which may, in turn, act as a trigger for re-entrant scar-mediated VT.

Thus, treatment with antiparasitic medication may lead to a decrease in the inflammation seen on 18-FDG PET and, consequently, a decrease in the incidence of potentially lethal ventricular arrhythmias.

Moreover, the patient was on amiodarone prior to receiving antiparasitic therapy. It has been postulated that amiodarone has anti–*T cruzi* activity, with animal studies demonstrating disruption of Ca^2+^ homeostasis and blocking oxidosqualene-cyclase enzyme, causing structural damage.^7^^,^^8^ Subgroup analysis from the BENEFIT trial found a significant reduction in the primary composite endpoint in those on amiodarone and benznidazole compared with benznidazole alone. However, rates of parasite detection polymerase chain reaction between the benznidazole groups were similar regardless of the receipt of amiodarone at baseline in the BENEFIT trial and other studies.^5^^,^^9^ Although it is possible that amiodarone played a role in decreasing the rate of ventricular arrhythmias, the exact mechanism is unknown, and further research is required.

To our knowledge, this is the first case report to use antiparasitic treatment in a patient with recurrent ventricular arrhythmias with correlating 18-FDG PET imaging. These findings need to be replicated on a larger scale for further analysis, but have potentially significant ramifications for the treatment of CD. The current treatment guidelines only suggest treatment for a select group of patients with CD based on the BENEFIT trial,^5^ but these results could potentially widen that group to include those with ventricular arrhythmias and inflammation on 18-FDG PET.

### Limitations

Inferences drawn from this report have several limitations. Firstly, it is only a singular case report, which limits generalizability of the findings. The abnormal inflammation observed on 18-FDG PET could be attributable to other conditions, including sarcoidosis, which was not definitively ruled out. Secondly, it remains unclear whether resolution of inflammation was due to antiparasitic treatment directly, given there was no immediate pre- and post-treatment 18-FDG PET conducted.

## Conclusion

This case report described a patient with CD and recurrent VT who underwent antiparasitic treatment with benznidazole, ultimately leading to resolution of cardiac inflammation and ventricular arrhythmias. This suggests that inflammation detected by PET-CT may play a role in VT in patients with CD and that antiparasitic therapy could be an effective treatment strategy, potentially expanding current treatment guidelines.

## Disclosures

Justin Hayase is a consultant for Anumana and Jason S. Bradfield received honoraria from Abbott and Atricure; the rest of the authors have no conflicts of interest.
